# Tuning the Solvation Structure in Water‐Based Solution Enables Surface Reconstruction of Layered Oxide Cathodes toward Long Lifespan Sodium‐Ion Batteries

**DOI:** 10.1002/advs.202401514

**Published:** 2024-05-02

**Authors:** Youchen Hao, Yufan Xia, Wen Liu, Guojie Sun, Lihua Feng, Xiaochong Zhou, Sikandar Iqbal, Ziqi Tian, Zhongcai Zhang, Yong Li, Xuan Zhang, Yinzhu Jiang

**Affiliations:** ^1^ School of Materials Science and Engineering Zhejiang University Hangzhou 310027 China; ^2^ Future Science Research Institute ZJU‐Hangzhou Global Scientific and Technological Innovation Center Zhejiang University Hangzhou 311215 China; ^3^ Tsinghua Shenzhen International Graduate School Tsinghua University Shenzhen 518055 China; ^4^ Huzhou Horizontal Na Energy Technology Co., Ltd. Huzhou 313000 China; ^5^ School of Physics and Materials Science Nanchang University Nanchang Jiangxi 330031 China

**Keywords:** layered oxides, sodium‐ion batteries, solvation tuning strategy, surface reconstruction, water‐based solution

## Abstract

Layered oxides of sodium‐ion batteries suffer from severe side reactions on the electrode/electrolyte interface, leading to fast capacity degradation. Although surface reconstruction strategies are widely used to solve the above issues, the utilization of the low‐cost wet chemical method is extremely challenging for moisture‐sensitive Na‐based oxide materials. Here, the solvation tuning strategy is proposed to overcome the deterioration of NaNi_1/3_Mn_1/3_Fe_1/3_O_2_ in water‐based solution and conduct the surface reconstruction. When capturing the water molecules by the solvation structure of cations, here is Li^+^, the structural collapse and degradation of layered oxides in water‐based solvents are greatly mitigated. Furthermore, Li(H_2_O)_3_EA^+^ promotes the profitable Li^+^/Na^+^ exchange to build a robust surface, which hampers the decomposition of electrolytes and the structural evolution upon cycling. Accordingly, the lifespan of Li‐reinforced materials is prolonged to three times that of the pristine one. This work represents a step forward in understanding the surface reconstruction operated in a water‐based solution for high‐performance sodium layered oxide cathodes.

## Graphical abstract

The solvation tuning strategy is proposed to promote the stability of Na‐based oxides in water‐based solutions and then construct a robust surface via surface chemical reactions. Here, the typically moisture‐sensitive NaNi_1/3_Mn_1/3_Fe_1/3_O_2_ maintain well, instead of pulverization, in the water‐based LiAc solution and achieve the simultaneously interfacial Li^+^/Na^+^ exchange, which prolongs the lifespan to 3 times as the original one.

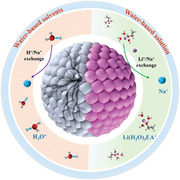

## 1 Introduction

Sodium‐ion batteries (SIBs) have been regarded as the most promising complementary alternatives to lithium‐ion batteries (LIBs) in the application of electrical grid energy storage and low‐speed electricity vehicles because of their low cost, natural abundance, and outstanding low‐temperature performances.^[^ ^1^ ^]^ However, developing high‐energy‐density SIBs that are competitive to LIBs is still limited, particularly by the “cask effect” of cathode materials.^[^ ^2^ ^]^ The layered oxides are most likely to provide high energy density but suffer from the severe capacity fading that is ascribed to structural deterioration and continuous Na loss.^[^ ^3^ ^]^ Generally, the gliding of transition metal (TM) layers arising from the electrostatic interaction among Na, O, and, TM may priorly lead to structural collapse on their surface given the dynamic inconsistency of layered oxides.^[^ ^4^, ^5^, ^6^, ^7^ ^]^ Moreover, the high‐soluble Na species with low Lewis acidity would induce the repeated generation of cathode‐electrolyte interphase and increased resistance,^[^ ^8^, ^9^ ^]^ which further aggravated the evolution of surface structure and the irreversible consumption of surface Na^+^. Therefore, regulating the surface structure is critically important in prolonging the lifespan of Na‐based layered oxides for practical application.

To date, numerous efforts have been engaged in designing the composition of layered oxides to mitigate their structural deterioration upon sodiation/desodiation.^[^ ^10^, ^11^, ^12^, ^13^ ^]^ Although most of them presented the suppressed phase change, the capacity fading was still serious which can be attributed to the continuous Na loss on their surface in long cycling.^[^ ^14^, ^15^, ^16^, ^17^ ^]^ Furthermore, it is found that the interaction between cations and oxygen is closely related to the interfacial stability of layered oxides.^[^ ^18^ ^]^ The Ni/Mn‐dominant oxides (Ni/Mn contents over 50% in the TM layer) with high TM─O bond covalency are normally unfavorable to tolerate the air and/or electrolytes. Therefore, endeavors of regulating the near‐surface structure with negligible capacity loss are necessary to resolve the aforementioned issues of high‐energy‐density Ni/Mn‐based oxides. The in situ chemistry reaction is believed to be an available way to build a functionalized surface, where the surface alkaline and/or TM sites of layered oxides can be reconstructed by the strong bindings of chemical reagents.^[^ ^19^, ^20^, ^21^, ^22^ ^]^ Cai and coworkers successfully rebuild the surface structure of LiCoO_2_ via an in situ Li^+^‐La^3+^/Ca^2+^ ion exchange reaction in an aqueous solution,^[^ ^23^ ^]^ the ultra‐thin surface (≈3 nm) not only blocks the direct contact of electrolytes/electrodes but also ensures the diffusion of Li^+^. More importantly, the in situ transformed interface with robust chemical bonds improved the structural integrity of layered oxides in the long run. Likewise, the in situ chelation reaction in aqueous solution also prolonged the lifespan of layered Li‐rich Mn‐based oxides due to the strong bindings between chelators and surface TM atoms.^[^ ^24^ ^]^ Although the employment of in situ chemical bonds is feasible to prolong the lifespan of layered oxides, the moisture‐sensitivity of Na‐based layered oxides arising from the typical H^+^/Na^+^ exchange blocks the application of in situ chemistry reactions in aqueous solution.^[^ ^25^, ^26^, ^27^ ^]^

Here, the solvation tuning strategy, taking the solvation structure of Li^+^ in a water‐based solution as an example, is proposed to decrease the activity of H_2_O and conduct the in situ Li^+^/Na^+^ exchange reaction for the surface reconstruction of O3‐type NaNi_1/3_Mn_1/3_Fe_1/3_O_2_ (NMF111) by virtue of the “pillar effect” of Li in the Na sites.^[^ ^28^ ^]^ The NMF111 was steadily maintained in the water‐based solution instead of pulverization with the assistance of the Li^+^ solvation structure. Moreover, the Li^+^/Na^+^ exchange was successfully achieved in the water/ethanol (W/EA) mixtures with 0.1 m LiAc because the Li(H_2_O)_3_EA^+^ structure with high solvation energy of −27.5 kcal mol^−1^ enables the desolvation of Li^+^. Finally, the robust surface of Li‐modified Na_1‐x_Li_x_Ni_1/3_Mn_1/3_Fe_1/3_O_2_ (≈2.5_mol_% Li) dramatically prevented the gradual phase variation from the surface to the bulk and gas emission from the decomposition of electrolyte, thus leading to enhanced reversibility (77.8% capacity retention after 500 cycles at 1 C) than the pristine one (54.1%) and prolonged lifespan from 150 to 450 cycles (80% capacity retention).

## 2 Results and Discussion

### 2.1 The Deterioration of O_3_‐NaNi_1/3_Mn_1/3_Fe_1/3_O_2_ in Water‐Based Solvent

**Figure 1:**
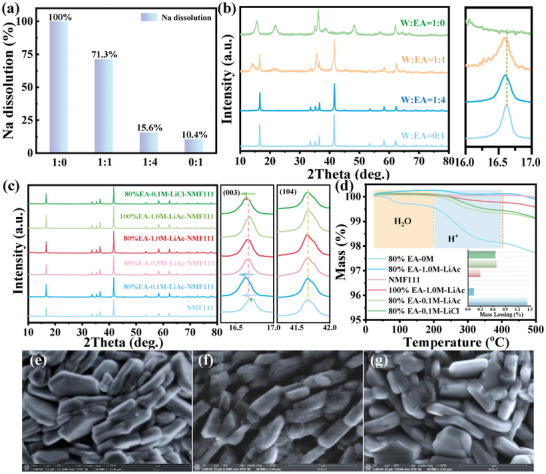
a) The ICP‐OES results of dissolved Na from NMF111 and b) the corresponding XRD patterns of the maintained NMF111 after soaking into different W/EA mixtures for 5 min. c) The XRD patterns and d) TG curves of NMF111 after soaking into different solutions for 5 min. SEM images of e) NMF111, f) 80%EA‐1.0m‐LiAc‐NMF111, and g) 100%EA‐1.0m‐LiAc‐NMF111.

The deterioration of layered oxide stems from the extraction of Na^+^ when exposed to wet air, and a more severe Na loss will occur in a water‐based solvent. Herein, various W/EA mixtures (v:v = 1:0, 1:1, 1:4, and 0:1, respectively) with a decreasing autoionization ability were utilized to testify the structural evolution of layered oxides upon soaking into these solvents. It was found that NMF111 was thoroughly destroyed in the water‐dominant solvent after soaking for 5 min, while the microscale sphere‐like particles were well maintained when the water ratio was less than 20% (Figure S1, Supporting Information). Meanwhile, ICP‐OES was also conducted to measure the Na^+^ dissolution in these solvents. The results in **Figure** **1**a show that all the Na^+^ escaped from NMF111 upon soaking in pure water within 5 min, accompanied by the collapse of layered oxide. On the contrary, there is only 15.6_mol_% of Na^+^ extracted from NMF111 in the 1:4 mixtures (marked as 80%EA), demonstrating the improved tolerance of NMF111 to water. Similarly, the corresponding XRD spectra of these soaked samples are displayed in Figure 1b, the characteristic signals belonging to the transition metal oxide agree to the thorough transformation of layered NMF111 when soaking in pure water. Whereas, although numerous Na^+^ (71.3_mol_%) have escaped from NMF111 in the 50%EA mixtures (W: EA = 1:1), the preserved layer structure proved the possibility of achieving the coexistence of water and NMF111.

### 2.2 The Regulation of Solvation Structure in Water‐Based Solution

Furthermore, the typical H^+^/Na^+^ exchange (Equation 1) was systematically confined by adjusting the coordination surroundings of water‐based solution in terms of the molecular dynamic,^[^ ^29^ ^]^ where both Li salt and solvent were utilized to restrict the activity of water.^[^ ^30^, ^31^, ^32^ ^]^ Besides, the crystalline structure of layer oxide was also enhanced due to the spontaneous Li^+^/Na^+^ exchange (Equation 2).

(1)

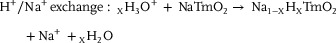

(2) $$\begin{array}{ccc} & & Li^{+}/Na^{+}\;\mathrm{exchange}:\;\mathrm{yL}i^{+}\;+\;\mathrm{NaTm}O_{2}\;\to \;Na_{1-y}Li_{y}\mathrm{Tm}O_{2}\\ & & \;+\;\mathrm{yN}a^{+} \end{array}$$

The details about the effect of solvents, Li salts, and their concentrations are discussed below:

First of all, high concentration LiAc solution (1.0 m) was selected to clarify the distinction between 80%EA and 100%EA solvents upon ion exchange (marked as 80%EA‐1.0m‐LiAc and 100%EA‐1.0m‐LiAc, respectively). Their overlapped XRD peaks in Figure 1c verified the limited H^+^/Na^+^ exchange in the water‐based solution. Meanwhile, a higher concentration of Li (1.96_mol_%) inside 80%EA‐1.0m‐LiAc‐NMF111 than the other (0.60_mol_%) showed the necessity of water for Li^+^/Na^+^ exchange (Figure S2, Supporting Information). Furthermore, the insertion of H^+^ inside these samples was detected by TG in Figure 1d, where the mass loss between 200–400 °C is regarded as the release of H^+^ from H^+^/Na^+^ exchange.^[^ ^33^ ^]^ Interestingly, there is about 1/10 of H^+^ (0.14_wt_%) remained in the samples soaked into 1.0 m LiAc solutions in contrast to 80%EA‐0m‐NMF111 (≈1.4_wt_%), demonstrating a further restricted H^+^/Na^+^ exchange after capturing free water by the solvation tuning strategy.

The morphologies of NMF111, 80%EA‐ and 100%EA‐1.0M‐LiAc‐NMF111 are given in Figure 1e–g. Differing from the rough surface of NMF111 that was immersed into the 80%EA solvent (Figure S1c, Supporting Information), all the modified NMF111 in 1.0 m LiAc solution retained a uniform surface, which corresponds to the limited H^+^/Na^+^ exchange inside these samples. Besides, the smooth surface of 100%EA‐1.0m‐LiAc‐NMF111 revealed a weaker Li^+^/Na^+^ exchange reaction than 80%EA‐1.0m‐LiAc‐NMF111 due to the absence of water. To intuitively understand the evolution of NMF111 after the insertion of H^+^ and Li^+^, the corresponding cycling performances of these materials are shown in Figure S3 (Supporting Information). Noticeably, although the crystalline structure and sphere‐like morphologies are well maintained, 80%EA‐0m‐NMF111 with the highest H^+^ content performed highest polarization and lowest capacity of ≈80 mAh g^−1^ at 0.1C (13 mA g^−1^). In reverse, the preserved reversibility of NMF111 in the LiAc solution illustrated the feasibility of manipulating the solvation structures. Moreover, 80%EA‐1.0m‐LiAc‐NMF111 with more Li^+^/Na^+^ exchange behaved the best performances than others. Therefore, it is believed that the spontaneous Li^+^/Na^+^ exchange promoted the structural stability of NMF111.

Afterward, the occurrence of H^+^/Na^+^ and Li^+^/Na^+^ exchanges is further evaluated by adjusting the concentration of Li salt to 0.1 and 0.5 m, respectively, in 80%EA solvent. It is found that NMF111 immersed in the low concentration (0.1 m) LiAc solution exhibited a slightly more (003) peak shift than the one in 0.5 and 1.0 m solutions (Figure 1c), which coincident well with the high H^+^ content of the former in Figure 1d, implying the decreased H^+^/Na^+^ exchange with the increasing of LiAc concentration. Meanwhile, the smooth surface of NMF111 is also maintained in the low‐concentration solutions (Figure S4, Supporting Information), even though the content of H^+^ in 80%EA‐0.1M‐NMF111 (0.69_wt_%) is ≈5 times as high as 80%EA‐1.0M‐NMF111, which different from the rough surface of 80%EA‐0M‐NMF111 without the protection of solvation structure. Moreover, the 80%EA‐0.1m‐LiAc‐NMF111 with more H^+^/Na^+^ exchange performed lower initial capacity but better stability than the one in higher concentration solutions (Figure S5, Supporting Information), which is ascribed to the formation of Na vacancies.^[^ ^14^, ^34^, ^35^ ^]^ Accordingly, the obvious distinction among morphologies, H^+^ content, and electrochemical performances of 80%EA‐0.1M‐NMF111 and 80%EA‐0M‐NMF111 reemphasized the critical role of solvation structure in the modification of NMF111, and the limited H^+^/Na^+^ exchange may help to construct a robust interface with appropriate Na vacancy and Li^+^ replacement.^[^ ^11^ ^]^

In addition, the anionic coordination may also contribute to regulating the H‐bond network and solvation structure.^[^ ^36^ ^]^ Meanwhile, the typical hydrolytic reaction of Ac^−^ is listed in Equation 3, which may hinder the corrosion of water as well (H^+^/Na^+^ exchange).

(3) $$H_{3}O^{+}\;+Ac^{-}\;↔\;\mathrm{HAc}\;+\;H_{2}O$$

Herein, both LiAc and LiCl are selected as the Li salt to explore the functions of anion to the H^+^/Na^+^ and Li^+^/Na^+^ exchange of NMF111 in 80%E solution. Apart from a slightly more Li^+^/Na^+^ exchange in the LiCl solution in Figure S2 (Supporting Information), both of them show similar morphologies, (003) peak shift and H^+^ insertion (Figure S4d, Supporting Information; Figure 1c,d). Nonetheless, the higher reversible capacity of 80%EA‐0.1M‐LiCl‐NMF111 in the initial several cycles illustrated that the anionic (Cl^−^) may strongly impact the solvation structure of Li^+^ in Figure S6 (Supporting Information), which is more important than the hydrolytic reaction of Ac^−^ on reducing the active H^+^ in water‐based solutions.

**Figure 2:**
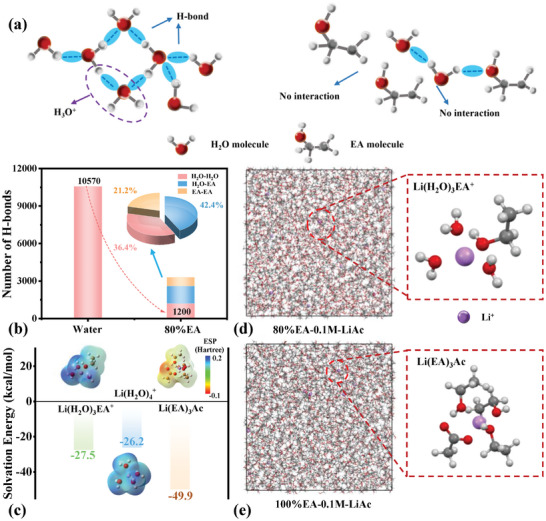
a) Schematic illustration of H‐bond inside pure water and 80%EA solution. b) The number of H‐bonds in pure water and 80%EA solution. The insets: the origins of H‐bond inside 80%EA solution and their proportions. c) The solvation energy of different solvation structures and their corresponding electrostatic potential (ESP) maps. The solvation structure of Li^+^ in various solutions: d) 80%EA‐0.1m‐LiAc and e) 100%EA‐0.1m‐LiAc solution.

The contributions from solvent and Li salts are further quantified via the molecular dynamic simulations in **Figure** **2**. Above all, the number of H‐bonds in pure water and 80%EA solution were calculated, respectively, because the dramatic H^+^/Na^+^ exchange of NMF111 mainly originated from the autoionization between H_2_O molecules (Figure 2a; Figure S7, Supporting Information). Evidently, the results in Figure 2b show that there is about 1/3 of H‐bonds remained in 80%EA solution in contrast to pure water, and only 36.4% of them come from the polar water molecules, which fits in well with their preserved sphere‐like particles and crystalline structure in Figure S1 (Supporting Information) and Figure 1b. Furthermore, the solvation structures of LiAc in 80%EA and 100%EA were also conducted to evaluate the function of H_2_O in Figure 2d,e, and Figure S8 (Supporting Information). The suppressed H^+^/Na^+^ exchange in 80%EA‐0.1m‐LiAc solution may account for the captured free water in Li(H_2_O)_3_EA^+^ solvation structure. Meanwhile, the solvation energy of Li(H_2_O)_3_EA^+^ and Li(EA)_3_Ac belonging to the solvation of 0.1 m LiAc in 80%EA and 100%EA solutions, respectively, were compared in Figure 2c, the lower solvation energy of latter (−49.9 kcal mol^−1^) than the former (−27.5 kcal mol^−1^) verified the weak Li^+^/Na^+^ exchangeability in the 100%EA‐0.1m‐LiAc solution. Lastly, the solvation effect of anionics was also compared in Figure 2c and Figures S8 and S9 (Supporting Information), where the fully Li‐O_W coordination and higher solvation energy of Li(H_2_O)_4_^+^ (−26.2 kcal mol^−1^) in LiCl solution caused the less Na loss of NMF111, therefore delivering higher initial capacity than the one in LiAc solution. From corresponding electrostatic potential maps of solvation structures (insert in Figure 2c), the decreased electrostatic potential can be observed as the solvation energy decreases. The solvation structures of Li(H_2_O)_3_EA^+^ and Li(H_2_O)_4_^+^ are relatively positive electrostatic potential values, which further confirms the easier desolvation process beneficial for Li^+^/Na^+^ exchange.

Therefore, it is concluded that the typical H^+^/Na^+^ exchange can be suppressed by manipulating the solvation structure, and the simultaneous Li^+^/Na^+^ exchange is profitable for a robust interface. This unique strategy of configuring the coordination environment of the solution is widely acceptable for the improvement of Na‐based oxides in a water‐based solution and has been verified by replacing the LiAc with KAc and CsAc respectively in Figure S10 (Supporting Information).

### 2.3 Surface Structure after Li^+^/Na^+^ Exchange

**Figure 3:**
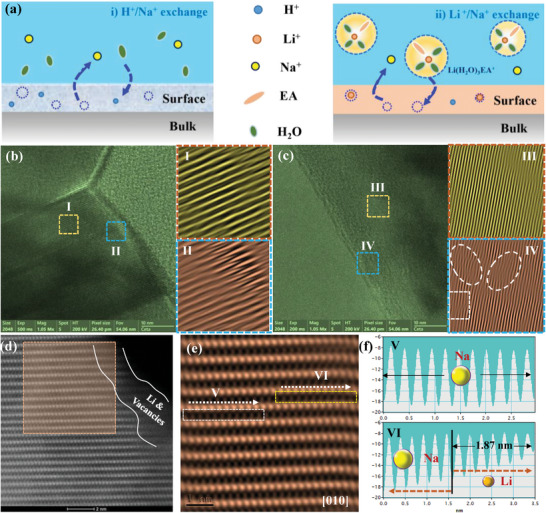
a) Schematic illustration of the ion exchange reactions on the surface of NMF111. High‐resolution TEM images of b) NMF111 and c) 80%EA‐0.1m‐5 min‐NMF111. The insets: the local magnification images in the I–IV areas. d) STEM‐HAADF image on the surface of 80%EA‐0.1m‐5 min‐NMF111. e) Enlarged view of the orange region marked in d). f) Intensity line profiles extracted along the white arrow in the V and VI regions, respectively.

The interfacial evolutions of O3‐type NaNi_1/3_Mn_1/3_Fe_1/3_O_2_ in aqueous solution are drawn in **Figure** **3**a. The obvious cracks on the surface of NMF111 (Figure 3b) are normally ascribed to the extraction of Na^+^ and the insertion of H_2_O when exposed to air,^[^ ^37^ ^]^ which is further verified by the EDS mapping in Figure S11 (Supporting Information). In detail, the corresponding local area presented more cracks on its surface, where Ni, Mn, Fe, and O elements are uniformly spread among the whole area, but the apparent Na voids can be observed in the crack region. In contrast, the characteristic lattice fringes are well maintained in the modified NMF111 (80%EA‐0.1m‐5 min‐NMF111) from bulk to surface, and only a slight lattice distortion appeared on its out‐surface, which may stem from the replacement of Na by Li/H with a smaller ionic radius (Figure 3c). To better understand the structural evolution on the surface of modified NMF111, the high‐resolution STEM was applied to detect the extraction of Na and the gliding of TMO_2_ slabs. Considering the orientation of Na^+^ diffusion in the Na‐based oxides, the surface structure along the (003) edges is detected in Figure 3d,e. Apparently, the intact TM layer revealed that the Li atoms only occupied the Na slab, even though the Li/Ni intermixing commonly occurred in previous Li‐modified cathodes.^[^ ^38^ ^]^ Furthermore, the atomic distribution on the Na slabs was conducted in Figure 3f, the weakened intensity on the near‐surface (VI region) illustrated that the light Li atoms and/or Na vacancy occupied the surface area with a thickness of ≈1.9 nm. Therefore, according to the interactions among Li^+^, H^+^, and Na^+^ in an aqueous solution discussed above, the Na vacancy and Li occupancy in the Na layer are believed the main reasons that resulted in the surface distortion, and the Li signals are further collected via XPS in Figure S12 (Supporting Information). Such a uniform interface is capable of driving the homogeneous Na^+^ diffusion and structural stability in the long run.^[^ ^28^, ^39^ ^]^

### 2.4 Enhanced Cyclability and Suppressed P3–O3 Irreversibility

Based on the results mentioned above, 0.1 m of LiAc in 80%EA solvent is confirmed as the optimal solution realizing the expected Li^+^/Na^+^ exchange and appropriate Na deficiency. Nonetheless, the continuous H^+^ insertion in such a low‐concentration solution may result in the failure of NMF111 because of the enlarged polarization. Thus, a variety of NMF111 was soaked into 80%EA‐0.1m‐LiAc solution for 2, 5, and 10 min, respectively, to evaluate the balance between Li‐reinforced interface and aggravated polarization. Interestingly, the 80%EA‐0.1m‐2 min‐NMF111 maintained a smooth surface after processing, while more tiny particles were observed in the materials soaked for a long time (Figure S13, Supporting Information). Likewise, the corresponding XRD patterns of these samples in Figure S14 (Supporting Information) presented a successive (003) peak shift to a low angle with the increase of immersion time, demonstrating the sustained H^+^/Na^+^ exchange in the low‐concentration solution.

**Figure 4:**
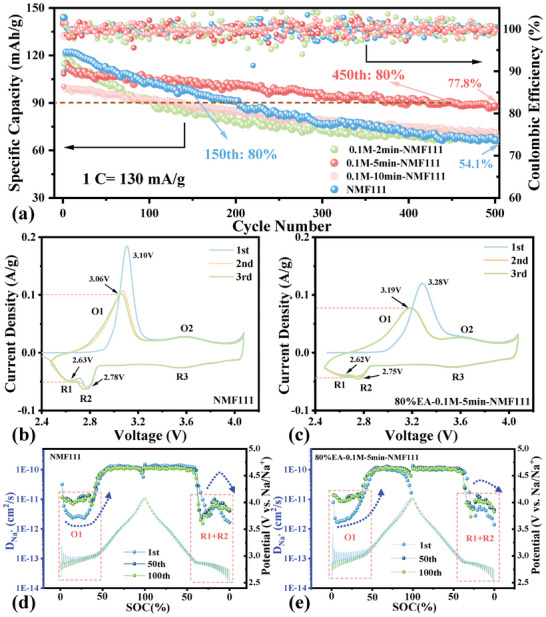
a) Long‐term cyclability of pristine and modified NMF111 soaked into 80%EA‐0.1m LiAc solution at various times. The CV curves and GITT profiles of b, d) NMF111 and c, e) 80%EA‐0.1m‐5min‐NMF111, respectively.

The long‐term cyclability of these materials is further detected in **Figure** **4**a to investigate the electrochemical behavior of NMF111 after Li^+^/Na^+^ exchange. Noticeably, the negative correlation between the initial capacity and immersion time coincident well with the continuous insertion of H^+^, and the improved stability of modified samples highlights the advantage of the Li^+^/Na^+^ exchange interface. The polarization of processed NMF111 is shown in Figure S15 (Supporting Information), the elevated charge plateau from 2.9 to 3.1 V confirmed the necessity of controlling H^+^ insertion. Benefiting from appropriate Na deficiency and Li^+^ substitution, 80%EA‐0.1m‐5min‐NMF111 delivered a high capacity of 87.8 mAh g^−1^ after 500 cycles at 1 C, which is superior to the pristine one (66.0 mAh g^−1^). In addition, the prolonged lifespan from 150 to 450 cycles at 80% capacity retention verified the significance of surface enhancement.

To better understand the functions of the Li^+^/Na^+^ exchange interface in promoting the cyclability of NMF111, the charge/discharge profiles of all samples are unfolded in Figure S16 (Supporting Information). A similar capacity fading is observed in the samples without/mild Li^+^/Na^+^ exchange interface. In reverse, both 80%EA‐0.1m‐5 min‐NMF111 and 80%EA‐0.1m‐10 min‐NMF111 revealed good reversibility after a sufficient Li insertion. Because all the samples performed good reversibility within 3.3–4.1 V, the great distinction for NMF111 with/without Li^+^/Na^+^ exchange interface is highly relied on the stability within 3.0–3.3 V upon charging. CV curves of all samples are further conducted to verify the reversibility of various redox pairs in Figure 4b,c, and Figure S17 (Supporting Information). Their increasing oxidation peaks from 3.1 to 3.4 V in the initial cycle are ascribed to the intensive H^+^ insertion, agreeing to the decreased capacity and enlarged polarization in Figure S15 (Supporting Information). Particularly, the overlapped O2/R3 and unstable O1/(R1+R2) pairs are in accordance with the consequents of charge/discharge profiles. As reported before, the O1/(R1+R2) pair originated from the phase change from O3 to P3.^[^ ^34^, ^40^ ^]^ It is deduced that the Li^+^/Na^+^ exchange interface may enhance the structural stability, thus retarding the deterioration of NMF111 from the surface to bulk.

GITT in various cycles was applied to describe the structural evolution of NMF111 and 80%EA‐0.1m‐5 min‐NMF111 upon sodiation/desodiation (Figure 4d,e). Commonly, the Na diffusivity (D_Na_^+^) in the Na‐poor area (75%SOC, SOC is “state of charge”) is almost 2 magnitudes as high as in the Na‐rich region (25% SOC), which reveals the obvious distinction in the dynamic of P3 and O3 phases upon the initial cycle. Therefore, the phase change between P‐ and O‐type oxides is believed the main challenge resulting in the capacity loss of NMF111. Meanwhile, more details about the evolution of D_Na_^+^ in NMF111 and 80%EA‐0.1m‐5 min‐NMF111 are unfolded in Figure S18 (Supporting Information). Both of them presented a gradually increased D_Na_^+^ value upon charging, but the sudden drop from 10^−10^ to 10^−12^ cm^2^ s^−1^ upon discharging demonstrated the insufficient reversibility from P3 to O3 phase. However, the NMF111 with Li^+^/Na^+^ exchange interface remained a smooth transformation from P3 to O3 phase after a long run, illustrating the inhibited TM slab gliding with the insertion of Li into Na slabs.

**Figure 5:**
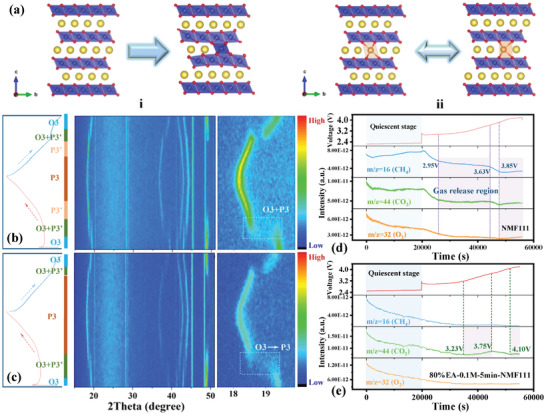
a) Schematic illustration of the structural evolution of (i) NMF111 and (ii) 80%EA‐0.1m‐5 min‐NMF111 upon cycling. in situ XRD spectrum and operando DEMS of b, d) NMF111 and c, e) 80%EA‐0.1m‐5 min‐NMF111, respectively, in the initial cycle.

More strikingly, in situ, XRD was carried out to study the changes between O3 and P3 phase upon the whole cycling, particularly in the transition region from 25%SOC to 75%SOC in GITT, where the typical (003) peak shift arising from the extraction of Na represented the gliding of TM slab and reciprocal transformation between O3 and P3 phase. As shown in **Figure** **5**b,c, the Li‐modified NMF111 went through a gradual transformation from the O3 to P3 phase with the extraction of Na, while both O3 and P3 phase coexist in NMF111 at the beginning of charging (25–50%SOC), illustrating the unstable structure of latter upon cycling. Besides, the persistent O3 phase from 0 to 25%SOC conformed with the lower D_Na_^+^ value of NMF1111 in Figure 4d. A similar process was also detected via ex situ XRD in Figure S19 (Supporting Information). Apart from the typical O3 to P3 phase change within the initial 8 h, the peak shift of (104) and (015) belonging to O3 and P3 phase, respectively, at high SOC were simultaneously observed, where the unstable P3 phase in NMF111 may result from the migration of TM to the Na layers upon charging (Figure 5a).^[^ ^41^, ^42^ ^]^ Therefore, the unsatisfactory performances of NMF111 are ascribed to its fragile structure that goes through the phase change of O3—O3+P3’—P3’—P3, which is prone to create the inactive phase. In contrast, the Li‐modified NMF111 quickly responded to the escaping of Na^+^ upon changing, which may result from the pillar effect of Li in the Na slabs. Moreover, the O3‐type 80%EA‐0.1m‐5 min‐NMF111 transformed to the P3 phase at the beginning of charging and was well maintained until the end. As a result, instead of back to the P3’ phase induced by the TM migration, the steady P3 phase demonstrated that the prolonged cyclability of Li‐modified NMF111 is contributed to its less lattice strain from O3 to P3 phase (O3—O3+P3’—P3) than the pristine one.

Apart from the structural enhancement, the tolerance of Li‐reinforced interface to the electrolyte is also compared via operando differential electrochemical mass spectrometry (DEMS) in Figure 5d,e and Figure S20 (Supporting Information). The increasing H_2_, CH_4_, and CO_2_ of NMF111 in quiescent stage demonstrated its sensitive surface to electrolyte than the modified NMF111. In addition, the production of H_2_ is regarded as the reduction of H_2_O,^[^ ^43^ ^]^ the continuous H_2_ releasing of 80%EA‐0.1m‐5 min‐NMF111 since the beginning of charge may account for the H^+^/Na^+^ exchange upon synthesizing, while the same trend of NMF111 after 2.95 V can be ascribed to the absorption of trace H_2_O in air. Nevertheless, the evolvement of CO_2_ and CH_4_ originated from the decomposition of carbonate solvents,^[^ ^44^, ^45^ ^]^ it is clear that the synchronous CO_2_/CH_4_ emission of NMF111 at 2.95 V corresponded to the phase change from O3 to P3 phase. In reverse, the CO_2_ emission of Li‐modified NMF111 has been delayed to 3.23 V, and no obvious CH_4_ signal is detected. Likewise, the high‐voltage evolution of electrodes performed an even better compatibility between Li‐reinforced interface and electrolyte, which is increased from 3.85 to 4.1 V. Particularly, the production of O_2_ is always attributed to the anionic redox of oxide cathodes, the eliminated O_2_ of modified NMF111 within 2.5‐4.2 V fits in well with the results of in situ XRD.

### 2.5 Preserved Layered Structure after a Long Run

**Figure 6:**
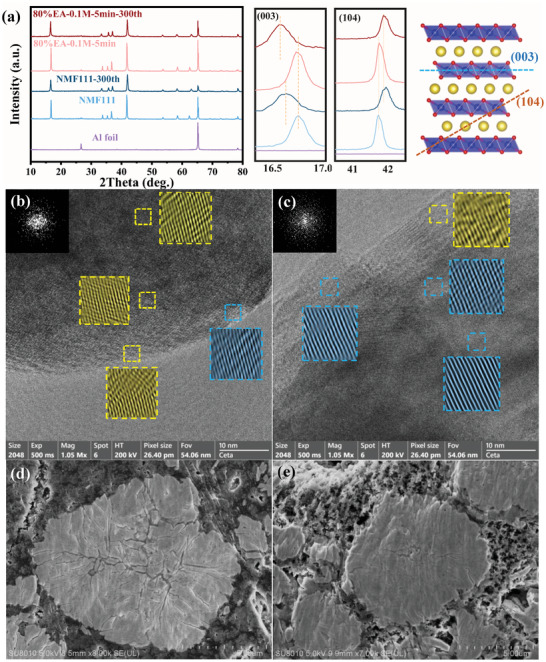
a) XRD patterns, b, c) TEM, and d, e) cross‐section images of NMF111 and 80%EA‐0.1m‐5 min‐NMF111, respectively, cycled at 1C for 300 cycles.

The long‐term structural evolution of pristine and modified NMF111 is also compared in **Figure** **6**. XRD patterns of electrodes are utilized to identify their reversibility after 300 cycles, both of them preserved the pure O3 phase even though the repetitive O3–P3 phase transition has occurred in each cycle. Nevertheless, it is inevitable that a slight peak shift can be observed because of less Na reinsertion and structural irreversibility. The (003) reflection represents the variation of d‐spacing, which is highly sensitive to the loss of Na. As is shown in Figure 6a, 80%EA‐0.1m‐5 min‐NMF111 exhibited more negative movement of (003) peak than NMF111, although the former achieved better reversibility than the latter. Indeed, the d‐spacing also relies on the interaction between Na─O and TM─O, the migration of TM can lead to an opposite movement of the (003) plane because of the weakened TM─O bonding.^[^ ^4^ ^]^ The greater (104) peak shift demonstrates a severe TM migration in the NMF111, therefore resulting in a less (003) shift in contrast to the modified materials. The lattice distortion resulting from the TM migration is further detected by TEM. The mixed diffraction spots and lattice fringe from surface to bulk testified the transformed structure from the layered to rock‐salt phase with cycling (Figure 6b). On the contrary, a clear lattice structure can be observed in Figure 6c, fitting in well with the results of in situ XRD and GITT that the Li^+^/Na^+^ exchange interface facilitates to enhance the stability of NMF111. More strikingly, the cross‐section images of cycled electrodes are shown in Figure S21 (Supporting Information) and Figure 6d,e. Because of the strong internal stress induced by the gliding of the TMO_2_ slab, the universal cracks are widely spread inside the secondary particles of NMF111, but only a few cracks can be observed in the core of 80%EA‐0.1m‐5 min‐NMF111, testifying the protection effect of Li^+^/Na^+^ exchange interface.

## 3 Conclusion

In summary, a functionalized Li^+^/Na^+^ exchange interface has been successfully fabricated on the surface of moisture‐sensitive O3‐type oxides by tuning the solvation structure of an aqueous solution. Typically, both the mixed solvent and the solvation structure prevented the severe H^+^/Na^+^ exchange by disrupting the H‐bond network and capturing free water, respectively, therefore maintaining the reversibility of oxides after being immersed into aqueous solutions. Furthermore, the characteristic Li(H_2_O)_3_EA^+^ solvation structure with high solvation energy of −27.5 kcal mol^−1^ achieved a simultaneous Li^+^/Na^+^ exchange in 80%EA‐0.1m‐LiAc solution, which helps to strengthen the structural stability of cathode materials and retard the gas emission from the side reaction between electrode and electrolyte. In addition, it is found that the occupations of Li are located in the Na sites, which successfully mitigates the transformation of TM upon cycling. Accordingly, the Li‐reinforced NMF111 went through a suppressed phase change of O3—O3+P3’—P3 in contrast to the pristine one (O3—O3+P3’—P3’—P3) and enabled the prolonged lifespan from 150 to 450 cycles at the capacity retention of 80%. The solvation strategy provides an available way for the design of fruitful interfaces of the most common oxides.

## 4 Experimental Section

### Creation of Li‐Modified NMF111

The O3‐type NaNi_1/3_Mn_1/3_Fe_1/3_O_2_ (NMF111) was provided by Horizontal Na Energy (Huzhou Horizontal Na Energy Technology Co., Ltd., Huzhou, China), which follows the routine carbonate co‐precipitation method. The Li‐modified NMF111 was fabricated via an ion‐exchange reaction in aqueous solution. Taking the fabrication of 80%EA‐0.1m‐5 min‐NMF111 as an example, 10.2 mg of LiAc was added into 1 mL of 80%EA solution and kept stirring until fully dissolved. Then the as‐prepared NMF111 (2 mmol) was immersed into this solution for 5 min at room temperature and then filtered immediately. Finally, the 80%EA‐0.1m‐NMF111 can be obtained by washing with ethanol several times and then drying at 80 °C for 3 h. The control groups can be fabricated following the same process via adjusting the components of solvent (80% or 100%EA), the concentrations of LiAc (0.1, 0.5, and 1.0 m), the immersion time (2, 5, and 10 min) and Li salts (LiAc or LiCl).

### Characterizations

The dissolved Na and insertion of Li elements were calculated by an inductively coupled plasma optical emission spectrometer (ICP‐OES, Agilent 5110). X‐ray diffraction (XRD) patterns were performed on an X‐ray diffractometer (Bruker D2 phaser, Germany) with Cu Kα radiation (= 1.5406 Å) at 30 kV, 10 mA. Thermogravimetric analysis (TGA, Discovery TGA 550) was utilized to probe the content of H^+^, which was operated in an N_2_ atmosphere with a heating rate of 10 °C min^−1^ from room temperature to 500 °C. The micro‐morphology and surface structure of samples were detected by scanning electron microscope (SEM, Thermofisher Scios2 Hivac, US) and high‐resolution transmission electron microscope (HR‐TEM, Talos F200X, US) with additional energy‐dispersive spectroscope (EDS). The atomic dispersion of the modified sample was performed in HAADF‐STEM mode, which was conducted on a Titan ChemiSTEM electron microscope operated at 200 KeV. The X‐ray photoelectron spectroscopy (XPS, Thermo Scientific K‐Alpha) measurements were performed to identify the insertion of surface Li elements.

For ex situ measurements, the electrodes at different charge/discharged states were disassembled in a glove box and washed with ethyl methyl carbonate (EMC) to remove residual electrolytes. The cross‐section images of cycled electrodes were detected after processing by a cross‐section polisher (CP, Fischione 1061 SEM Mill). In situ, XRD (Bruker D8 Advance with Co Kα radiation) measurements were performed in the initial cycle at 0.1 C, and the electrodes were assembled in a special device (purchased from the Beijing Scistar Technology Co. Ltd. China) with a beryllium window for X‐ray penetration. Each curve was collected at 2Theta ranging from 15 to 53° at a scanning rate of 0.04° s^−1^.

### Differential Electrochemical Mass Spectrometry (DEMS)

The *operando DEMS* (Shanghai Pro‐tech Co. Ltd. China) measurements were utilized to trace the gas environment inside batteries upon cycling. The battery mold was first assembled in the glove box, where the Na metal was the anode and the electrodes as cathode. Then, the batteries were operated in the Neware system within 2.5–4.2 V at 0.2 C. Meanwhile, the gases inside the battery mold will be continuously taken by Ar with the flow of 0.5 mL min^−1^ and finally detected by DEMS.

### Quantum Chemistry Calculations

Quantum chemistry calculations were performed using the Gaussian 16 software package (Revision C.01).^[^ ^46^ ^]^ The electronic structure of the systems under investigation was treated at the level of density functional theory (DFT) using the M06‐2X functional.^[^ ^47^ ^]^ The basis set employed was the def2‐TZVP basis set,^[^ ^48^ ^]^ which includes triple‐ζ quality basis functions with polarization and diffuse functions. The Grimme D3 dispersion correction^[^ ^49^ ^]^ was applied to account for long‐range dispersion interactions. To incorporate the solvent effects, the Polarizable Continuum Model (PCM)^[^ ^50^, ^51^ ^]^ was employed with ethanol as the solvent. In addition to geometry optimizations and frequency calculations, solvation energy calculations were performed using the PCM model to estimate the influence of the solvent on the energetics of the systems.

The solvation energy (*E*_sol._) was calculated using the following equation:

(4) $$E_{soL}\;=\;E_{\left\{Li{\left(H_{2}O\right)}_{x}{\left(EA\right)}_{y}{\left(Ac\right)}_{z}\right\}}\;-\;E_{Li^{+}}\;-\;E_{\left\{{\left(H_{2}O\right)}_{x}{\left(EA\right)}_{y}{\left(Ac\right)}_{z}\right\}}$$

Where $E_{\left\{Li{\left(H_{2}O\right)}_{x}{\left(EA\right)}_{y}{\left(Ac\right)}_{z}\right\}}$ is the total energy of different solvation structures. $E_{Li^{+}}$ is the energy of the Li‐ion. $E_{\left\{{\left(H_{2}O\right)}_{x}{\left(EA\right)}_{y}{\left(Ac\right)}_{z}\right\}}$ is the energy of other coordination molecules or ions in a solvation structure.

### Molecular Dynamics Simulations

The molecular dynamics simulations were conducted Forcite module in Materials Studio, employing the COMPASS III force field^[^ ^52^ ^]^ to elucidate the structural and dynamic behavior of the studied solution system. In the 100% ethanol solution, the composition is defined by the presence of 1713 ethanol molecules. Conversely, in the 80% ethanol + 20% H_2_O solution, the mixture consists of approximately 1371 ethanol molecules and 1110 water molecules. In the 100% H_2_O solution, there are 5550 water molecules exclusively. All the simulated component ratios are consistent with the experiment. A combination of optimization algorithms was first employed, comprising steepest descent, adopted basis Newton‐Raphson (ABNR), and quasi‐Newton methods. The convergence criteria for geometry optimizations were set at a maximum tolerance of 0.001 kcal mol^−1^ for the total energy and 0.5 kcal mol^−1^ Å^−1^ for the force. To equilibrate the simulation system, NPT (constant number of particles, pressure, and temperature) ensemble simulations using the Andersen thermostat^[^ ^53^ ^]^ for temperature control and the Berendsen barostat^[^ ^54^ ^]^ for pressure control. The simulations were run for a duration of 500 ps. For further conduct of the data collection, NVT (constant number of particles, volume, and temperature) ensemble simulations were performed utilizing the Nosé–Hoover thermostat^[^ ^55^, ^56^ ^]^ and were also executed for a total of 500 ps. A 1 fs time step was employed for all dynamics simulations. The long‐range electrostatic interactions were calculated using the Ewald scheme and atom‐based van der Waals interactions with a cutoff distance of 12.5 Å.

### Electrochemical Tests

The electrochemical performances of active materials were tested in the CR2032 coin cell. Typically, cathodes were fabricated by mixing 80wt.% of active materials, 10wt.% of carbon black and 10wt.% of polyvinylidene difluoride (PVDF) binder as well as appropriate 1‐Methyl‐2‐pyrrolidinone (NMP) as a homogenous slurry, and then cast on aluminum foil and dried in vacuum at 120 °C for 12 h, the mass loading of active materials for each electrode was about 2 mg cm^−2^. Na metal and glass fiber (GF/D) were employed as counter/reference electrodes and separators, respectively, to assemble the 2032 half‐cells in an argon‐filled glovebox, where the H_2_O and O_2_ were <0.01 ppm. 1 m NaPF_6_ in the mixture of propylene carbonate (PC), ethyl methyl carbonate (EMC), and fluoroethylene carbonate (FEC) additive with a volume ratio of 50:45:5 was adopted as electrolyte. The galvanostatic intermittent titration technique (GITT) and galvanostatic charge/discharge performance were evaluated on a Neware battery testing system in the potential range of 2.5–4.1 V (vs Na^+^/Na) at 25 °C. The GITT tests were conducted at a constant current of 0.1 C (13 mA g^−1^) for 900 s followed by a relaxation of 2700 s within the voltage window of 2.5–4.1 V. Cyclic voltammetry (CV) curves were recorded on a Princeton Applied Research VersaSTAT 4 electrochemical workstation.

## Conflict of Interest

The authors declare no conflict of interest.

## Author Contributions

Y.H. and Y.X. contributed equally to this work. Y.‐C.H., Y.‐F.X., and Y.‐Z.J. conceived the study and analyzed the results; Y.‐C.H. conducted the experiments, collected data, and wrote the manuscript; Y.‐F.X. conducted the MD and QC calculations; W.L. helped with cross‐section measurements and draw the crystalline structure; G.‐J.S., L.‐H.F., and S.I. helped with in situ XRD characterization; X.‐C.Z., Z.‐Q.T., and Z.‐C.Z. provided the pristine samples; Y.‐C.H., Y.‐F.X., Y.L., X.Z., and Y.‐Z.J. participated in the discussion of the data; Y.L., X.Z., and Y.‐Z.J. supervised the project. All authors have approved the final version of the manuscript.

## Supporting information

Supporting Information

## Data Availability

The data that support the findings of this study are available in the supplementary material of this article.
