# Supplementary material for: Tuning the Solvation Structure in Water‐Based Solution Enables Surface Reconstruction of Layered Oxide Cathodes toward Long Lifespan Sodium‐Ion Batteries
Source: Adv Sci (Weinh). 2024 May 2;11(26):2401514. doi: 10.1002/advs.202401514 (PMC11234404; doi:10.1002/advs.202401514)
Supplement: Supplementary file 1 — Supporting Information [file ADVS-11-2401514-s001.pdf]

ADVANCED SCIENCE
Open Access

Supporting Information

for *Adv. Sci.*, DOI 10.1002/advs.202401514

Tuning the Solvation Structure in Water-Based Solution Enables Surface Reconstruction of Layered Oxide Cathodes toward Long Lifespan Sodium-Ion Batteries

Youchen Hao, Yufan Xia, Wen Liu, Guojie Sun, Lihua Feng, Xiaochong Zhou, Sikandar Iqbal, Ziqi Tian, Zhongcai Zhang, Yong Li, Xuan Zhang* and Yinzhu Jiang**

Supplementary Information for

Tuning the Solvation Structure in Water-based Solution Enables Surface Reconstruction of Layered Oxide Cathodes toward Long Lifespan Sodium-ion Batteries

Youchen Hao,[1,2†] Yufan Xia,[1†] Wen Liu,[3] Guojie Sun,[1,2] Lihua Feng,[1,2] Xiaochong Zhou,[1] Sikandar Iqbal,[1,2] Ziqi Tian,[4] Zhongcai Zhang,[4] Yong Li,[5]* Xuan Zhang,[1,2]* Yinzhu Jiang[1,2]*

[1] School of Materials Science and Engineering, Zhejiang University, Hangzhou 310027, China

[2] Future Science Research Institute, ZJU-Hangzhou Global Scientific and Technological Innovation Center, Zhejiang University, Hangzhou 311215, China

[3] Tsinghua Shenzhen International Graduate School, Tsinghua University, Shenzhen 518055, China

[4] Huzhou Horizontal Na Energy Technology Co., Ltd., Huzhou, 313000, China

[5] School of Physics and Materials Science, Nanchang University, Nanchang, Jiangxi 330031, China

† These authors contributed equally to this work

* Corresponding authors: Yong Li: liyong1@ncu.edu.cn; Xuan Zhang: xuanzhangzju@zju.edu.cn; Yinzhu Jiang: yzjiang@zju.edu.cn

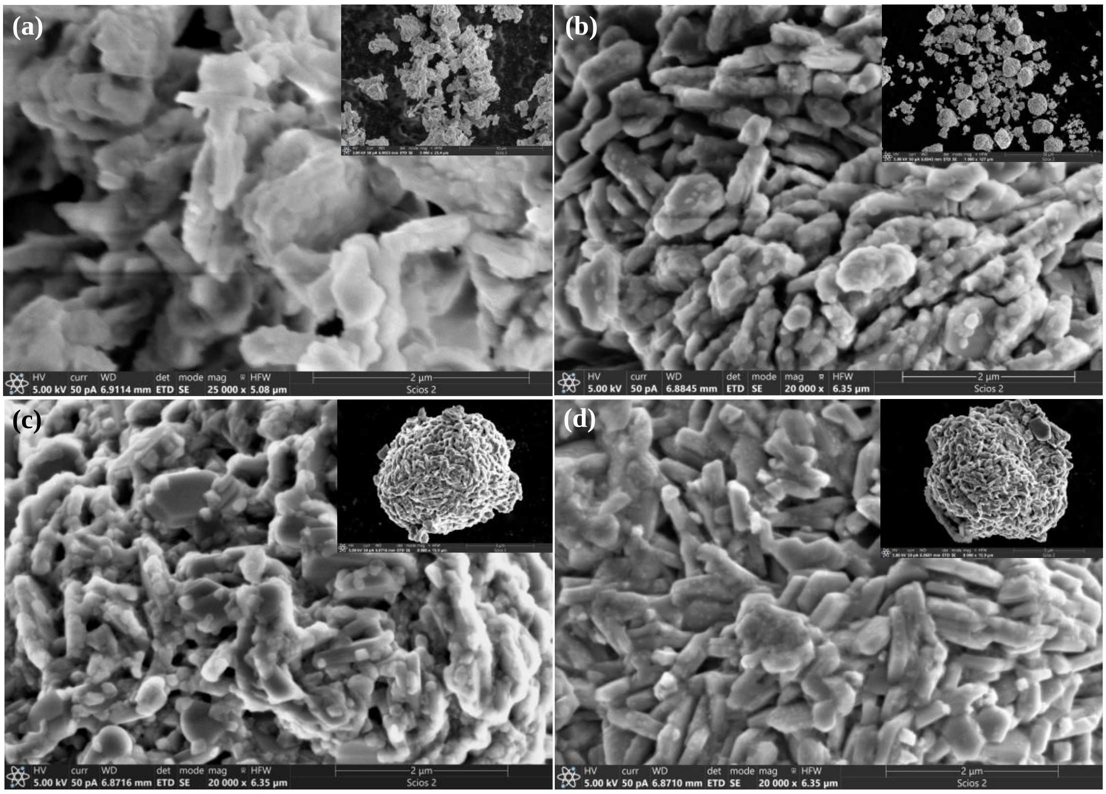

Figure S1 The morphologies of NMF111 after soaking into different solutions. High and low magnification SEM images of NMF111 after soaking into the W/EA mixture with the volume ratio of (a) 1:0, (b) 1:1, (c) 1:4, and (d) 0:1, respectively, for 5 min.

Supplementary Note 1. The moisture sensitivity of NMF111 is shown in Figure (a), its secondary particles have been thoroughly destroyed after immersing in pure water for 5min. However, the corrosion of NMF111 is greatly suppressed with the increasing ethanol content, which may result from the disturbed H-bond inside the solutions.

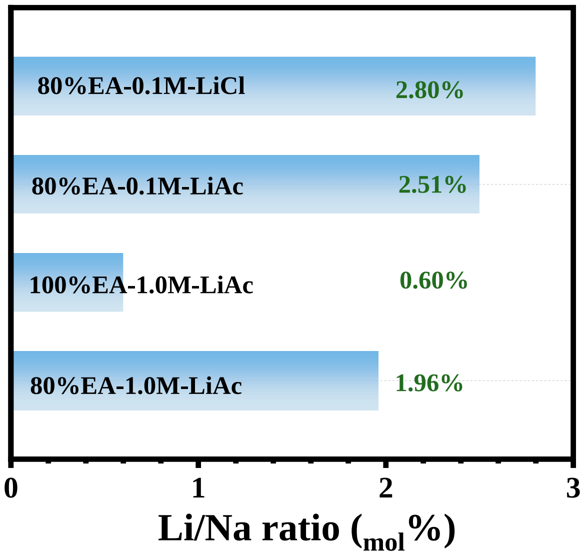

Figure S2 The Li/Na molar ratio of NMF111 after soaking into different solutions for 5 min.

Supplementary Note 2. Apart from the Li^+/Na^+ exchange, there are some Na^+ may be dissolved into the aqueous solution because of the simultaneous H^+/Na^+ exchange. Therefore, the accurate Li content in the modified NMF111 may be slightly lower than the Li/Na ratio, and it is believed that more H^+ is contained in the NMF111 when soaking into the low-concentration solution.

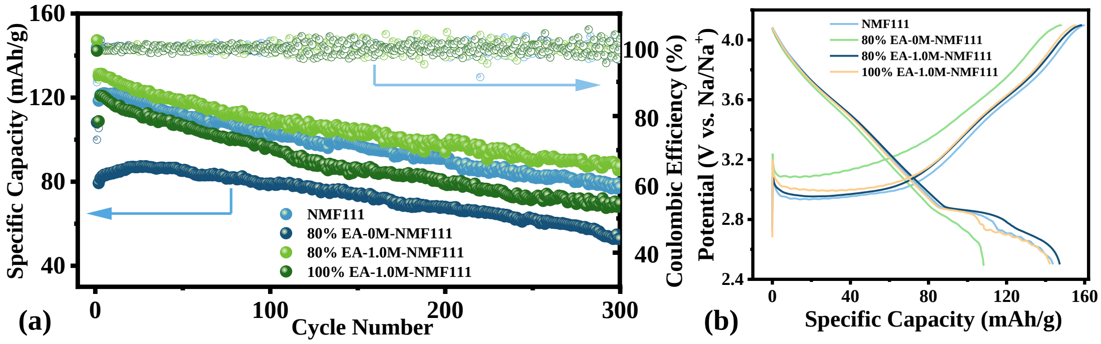

Figure S3 Cycling performances of NMF111 soaking into various solutions. (a) Long-term cycling performances and (b) the initial charge/discharge profiles of NMF111 after processing with 1.0M LiAc solutions.

Supplementary Note 3. Although the disruption of the H-bond network hampered the corrosion of NMF111 in a water-based solution, the worse cyclability of 80%EA-0M-NMF111 resulting from large polarization demonstrates the seriousness of H^+/Na^+ exchange. Fortunately, such polarization is easily resolved after the introduction of Li salts, which is ascribed to the coordination of Li^+ with water molecules. Moreover, 100%EA-1.0M-NMF111 with less H^+/Na^+ exchange performed worse cyclability than 80%EA-1.0M-NMF111, demonstrating the solvation structure in the former may be adversed to Li^+/Na^+ exchange.

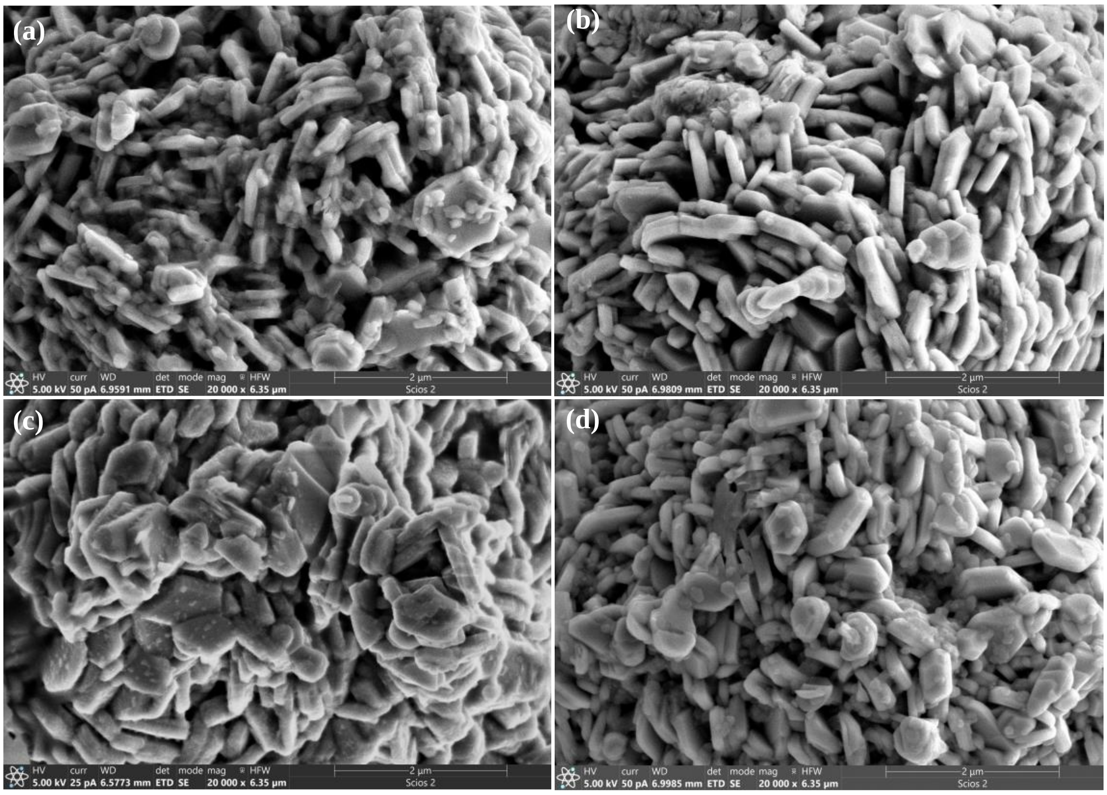

Figure S4 SEM images of NMF111 soaking into various solutions for 5 min: 80%EA- (a) 0.1M-LiAc, (b) 0.5M-LiAc, (c) 1.0M-LiAc and (d) 0.1M-LiCl.

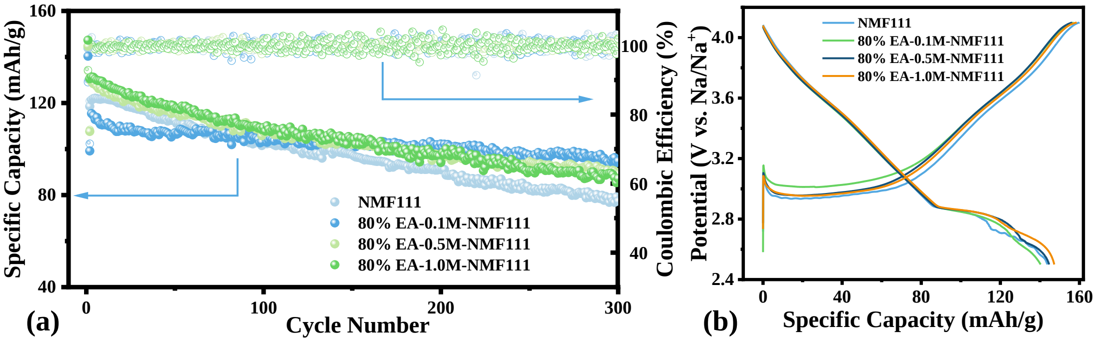

Figure S5 Cycling performances of NMF111 soaking into various concentration solutions. (a) Long-term cycling performances and (b) the initial charge/discharge profiles of NMF111 after processing in LiAc solutions with various concentrations for 5 min.

Supplementary Note 4. The solvation structure of the water-based solution facilitates anchoring the free water molecules, therefore hindering the H^+/Na^+ exchange of NMF111 upon soaking into aqueous solution. In addition, the high-concentration LiAc solution displayed more Li^+/Na^+ exchange, which may further inhibit the unfavorable H^+/Na^+ exchange. Consequently, all the materials immersed in 80%EA-LiAc solution maintained high performances in contrast to the one in 80%EA-0M solution. Furthermore, 80%EA-0.1M-NMF111 displayed better cyclability than the one in higher concentration LiAc solution, which may be ascribed to the formation of Na vacancies.

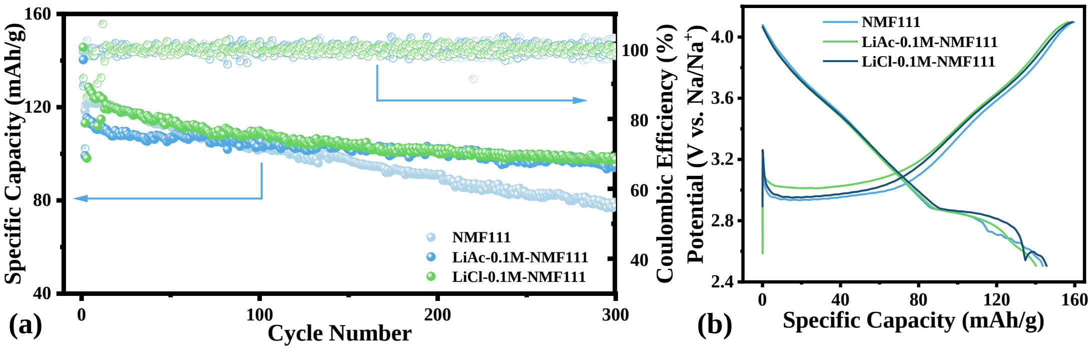

Figure S6 Cycling performances of NMF111 soaked into various Li salt solutions. (a) Long-term cycling performances and (b) the initial charge/discharge profiles of NMF111 after processing in 80%EA-0.1M LiAc and LiCl solutions, respectively, for 5 min.

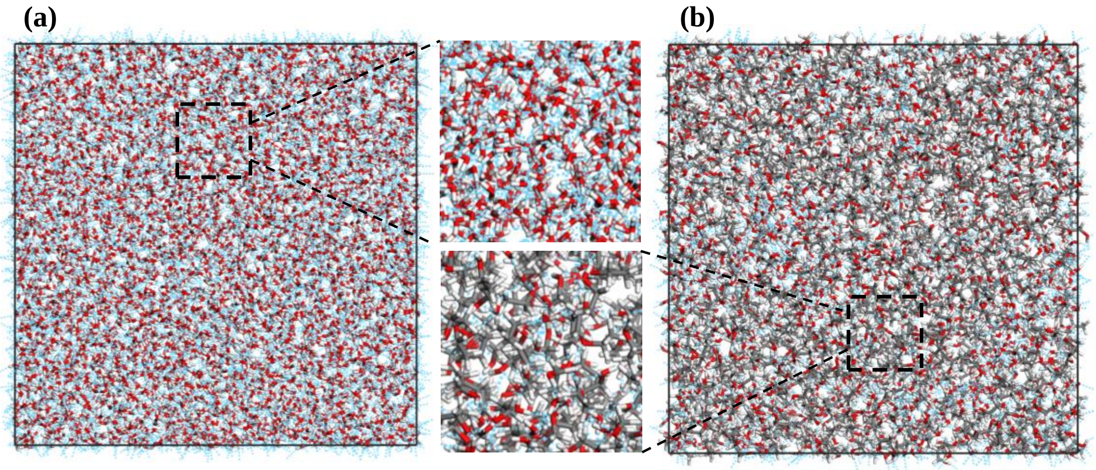

Figure S7 The calculated H-bonds inside (a) pure water and (b) 80%EA from MD simulations. The H-bonds are indicated by the blue dashed line.

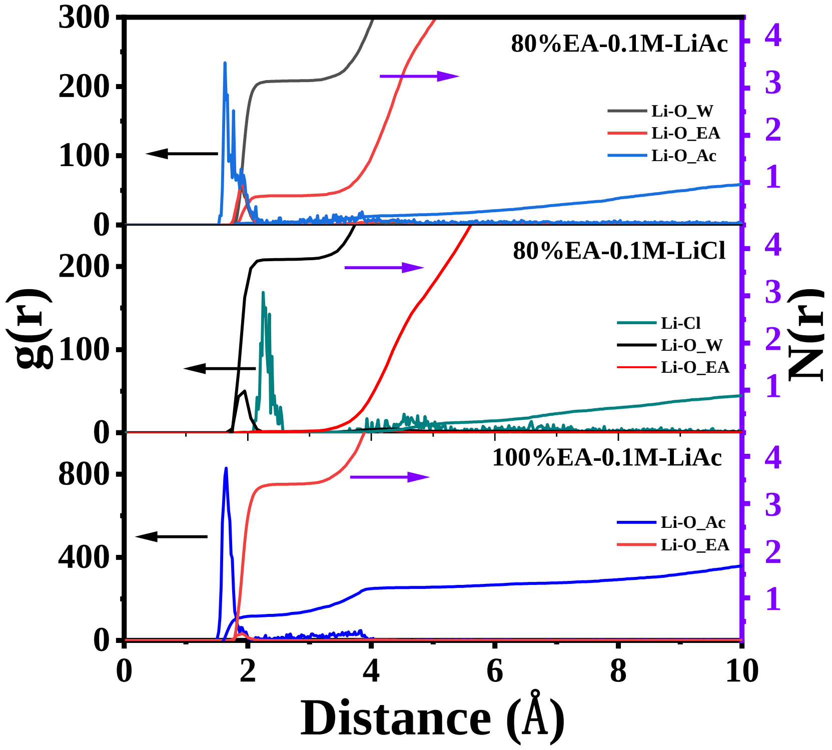

Figure S8 Radial distribution functions (RDFs) and the coordination number between Li-O/Cl in various solutions. O_W, O_EA, and O_Ac represent oxygen atoms in water molecules, ethanol molecules, and acetate ions, respectively.

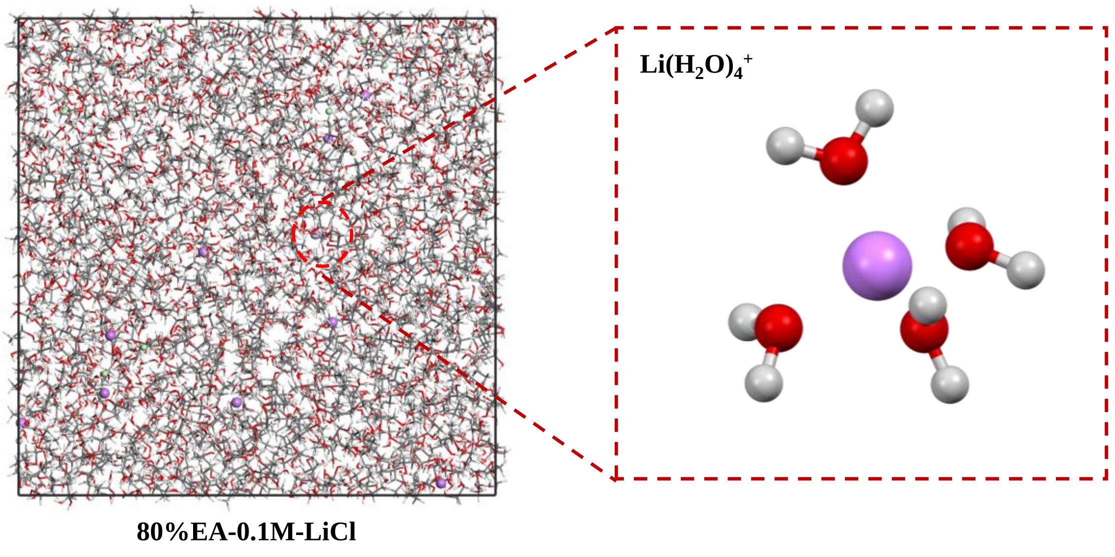

Figure S9 The snapshot and corresponding solvent sheath of 80%EA-0.1M-LiCl obtained from MD simulations.

Supplementary Note 5. Although the Cl^- did not participate in the coordination in the 80%-EA solution, the anionic induced the formation of $Li(H_2O)_4^+$ solvation structure. Consequently, a less H^+/Na^+ exchange and a higher initial cycle of 80%EA-0.1M-LiCl-NMF111 is observed than the one immersed in 80%EA-0.1M-LiAc solution.

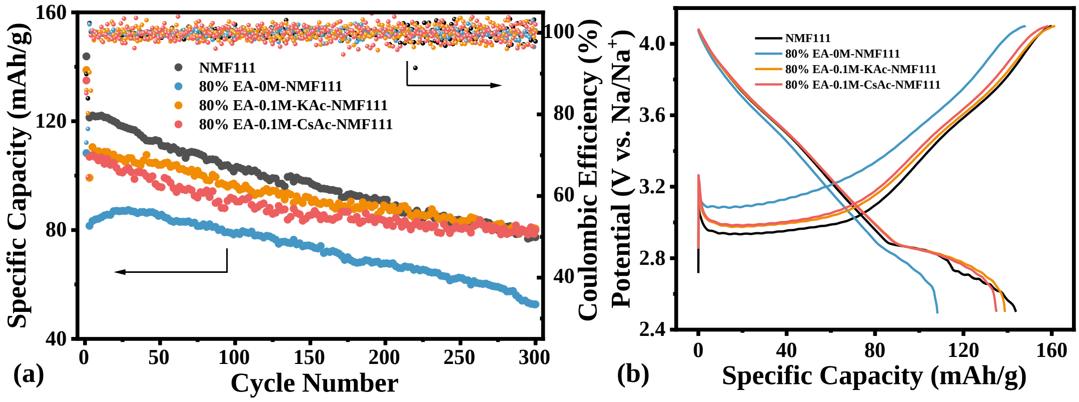

Figure S10 The cycling performances and corresponding charge/discharge profiles of NMF111 in 80%EA-0.1M-KAc and 80%EA-0.1M-CsAc solutions for 2min, respectively.

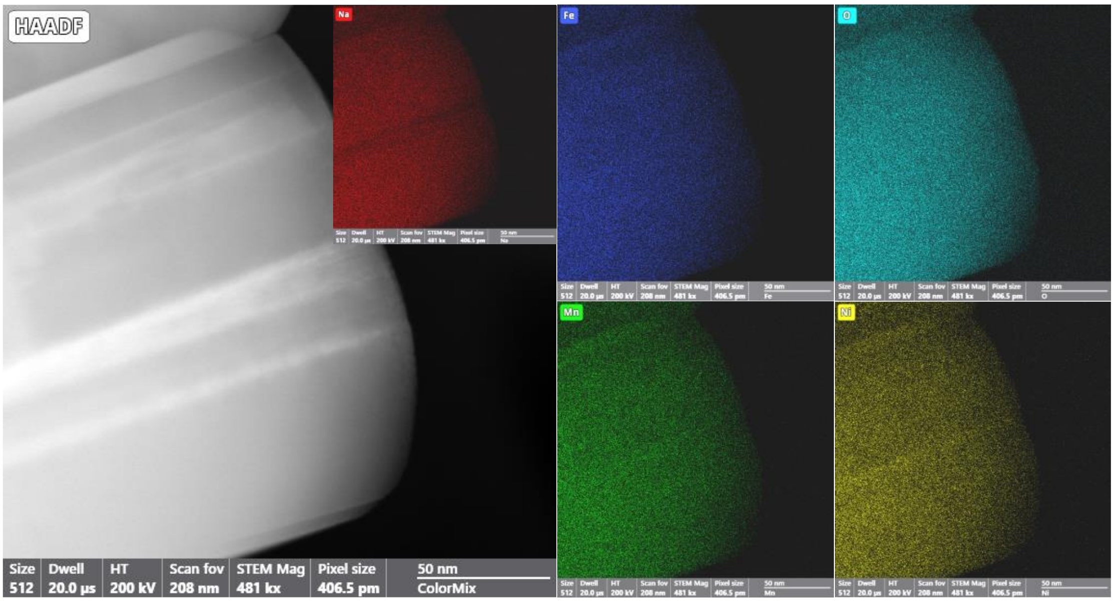

Figure S11 EDS mapping of NMF111.

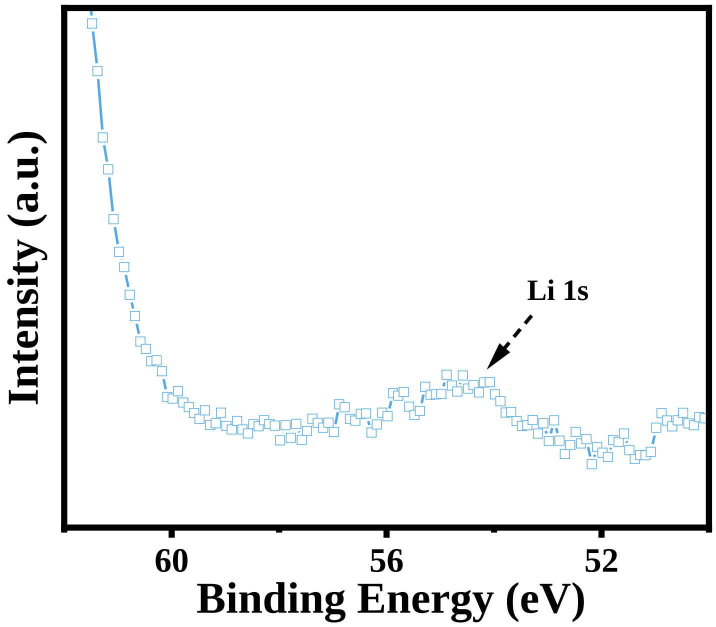

Figure S12 High resolution XPS Li 1s spectrum of 80%EA-0.1M-5min-NMF111.

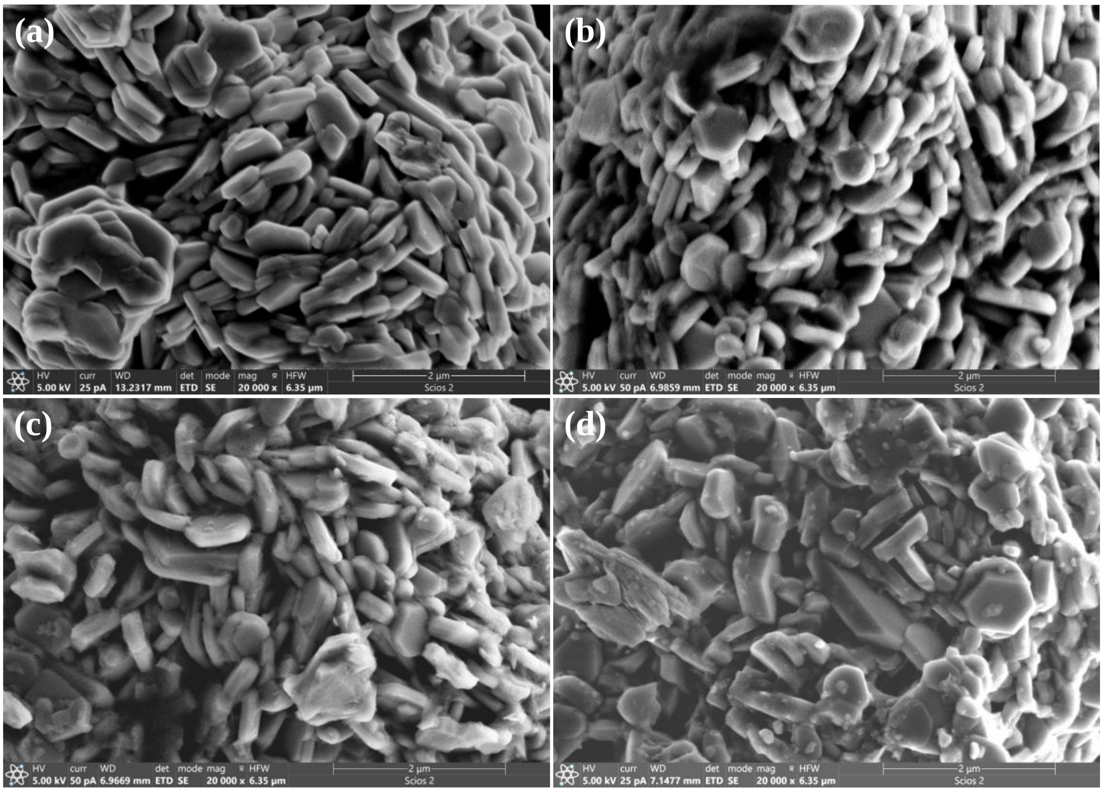

Figure S13 The morphologies of NMF111 immersed into 80%EA-0.1M-LiAc solution at various times. SEM images of (a) pristine and modified NMF111 soaking into 80%EA-0.1M for (b) 2, (c) 5, and (d) 10 min, respectively.

Supplementary Note 6. The low-concentration LiAc solution showed more H^+ insertion upon soaking into 80%EA solution, and more small particles were observed with the increase of immersion time.

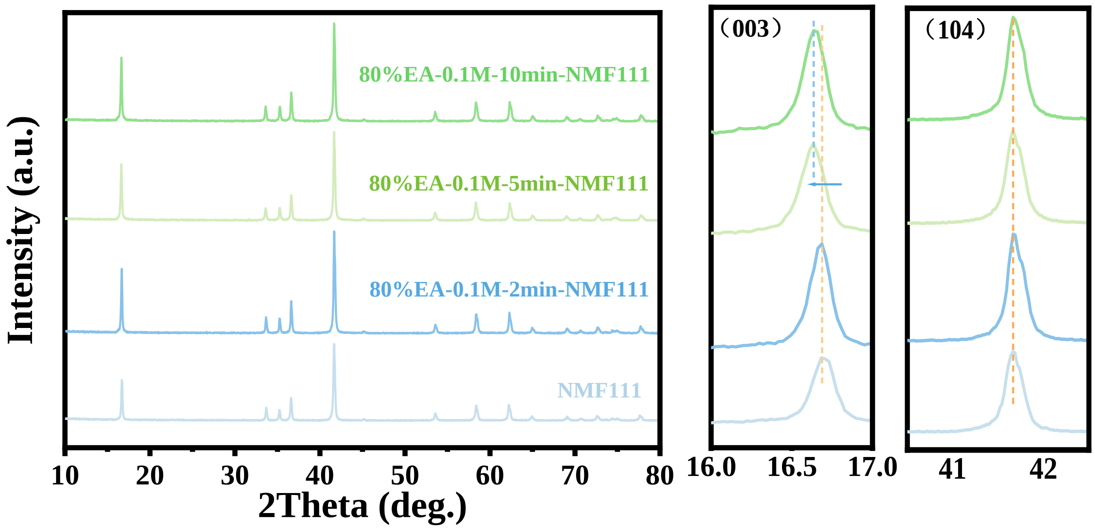

Figure S14 XRD patterns of pristine and modified NMF111.

Supplementary Note 7. The enlarged (003) peak shift demonstrated that the H^+ is continuously inserted into NMF111 upon soaking into 80%EA-0.1M-LiAc solution.

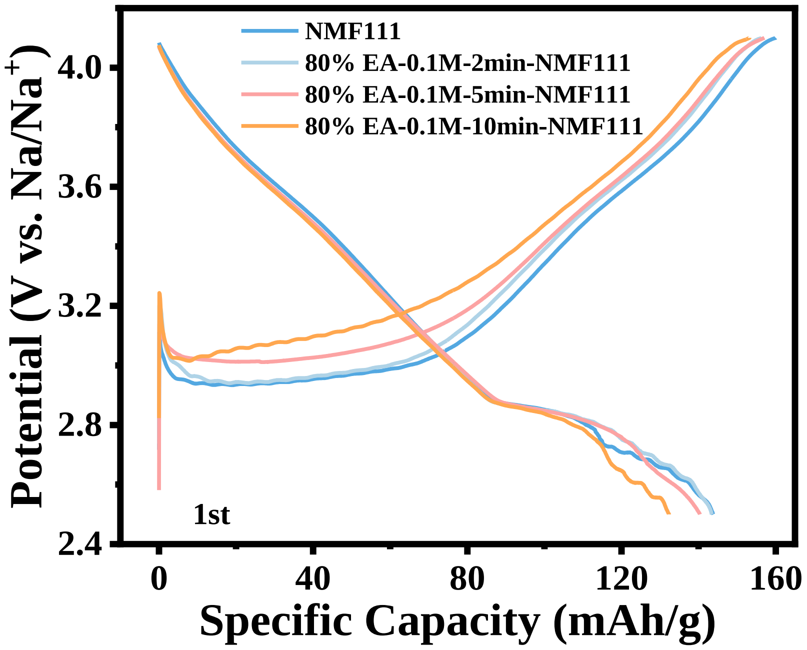

Figure S15 The charge/discharge profiles of pristine and modified NMF111 in the initial cycle at 0.1C.

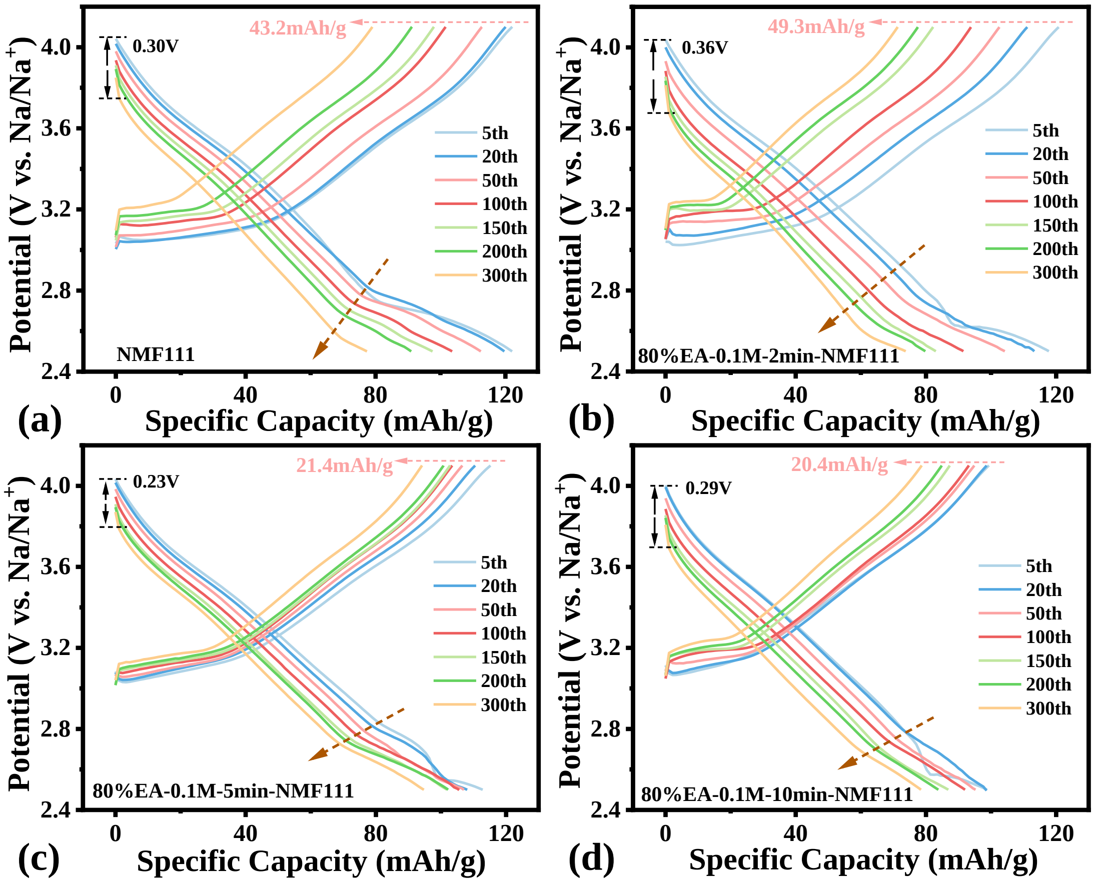

Figure S16 Charge/discharge profiles of all samples in the selected cycles at 1C.

Supplementary Note 8. The enhanced cyclability of 80%EA-0.1M-5min-NMF111 accounts for the reversible reactions at 3.1V upon charging, and all the samples preserved good reversibility at the voltage range from 3.4 to 4.1V.

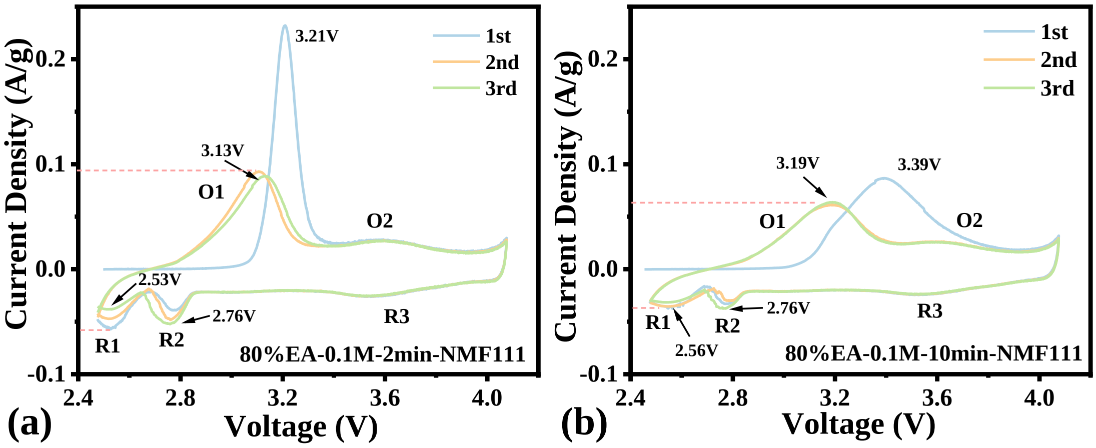

Figure S17 CV curves of NMF111 soaking into 80%EA-0.1M-LiAc for (a) 2 and (b) 10 min, respectively.

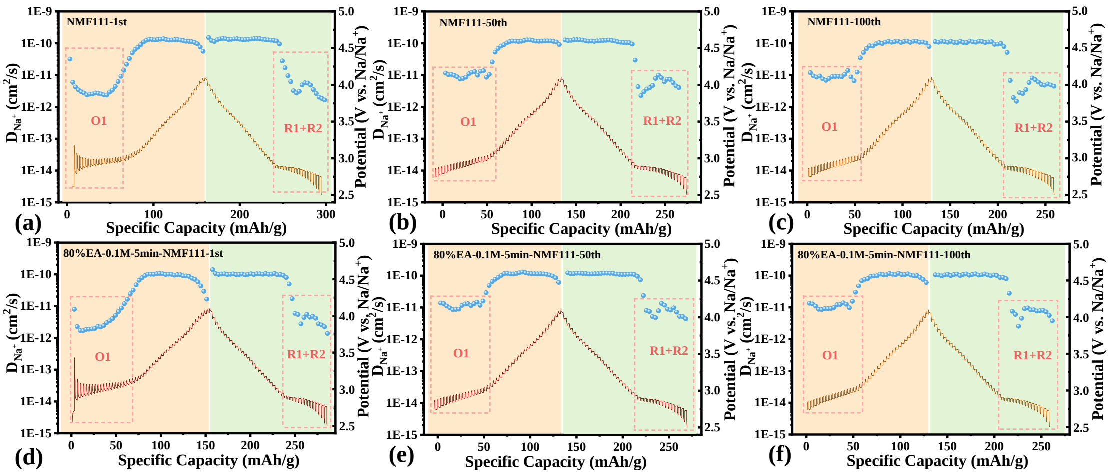

Figure S18 GITT profiles of NMF111 and 80%EA-0.1M-5min-NMF111 at the (a, d) 1st, (b, e) 50th, and (c, f) 100th cycle, respectively.

Supplementary Note 9. The worse reversibility of NMF111 belongs to the phase change between the O3 and P3 phase, and the enhanced cyclability of 80%EA-0.1M-5min-NMF111 is ascribed to the maintained structural stability from the P3 phase to the O3 phase. Typically, the D_{Na^+} value of NMF111 is suddenly decreased from 10^{-10} to 10^{-12} upon discharging, while the same process is decreased from 10^{-10} to 10^{-11} in 80%EA-0.1M-5min-NMF111.

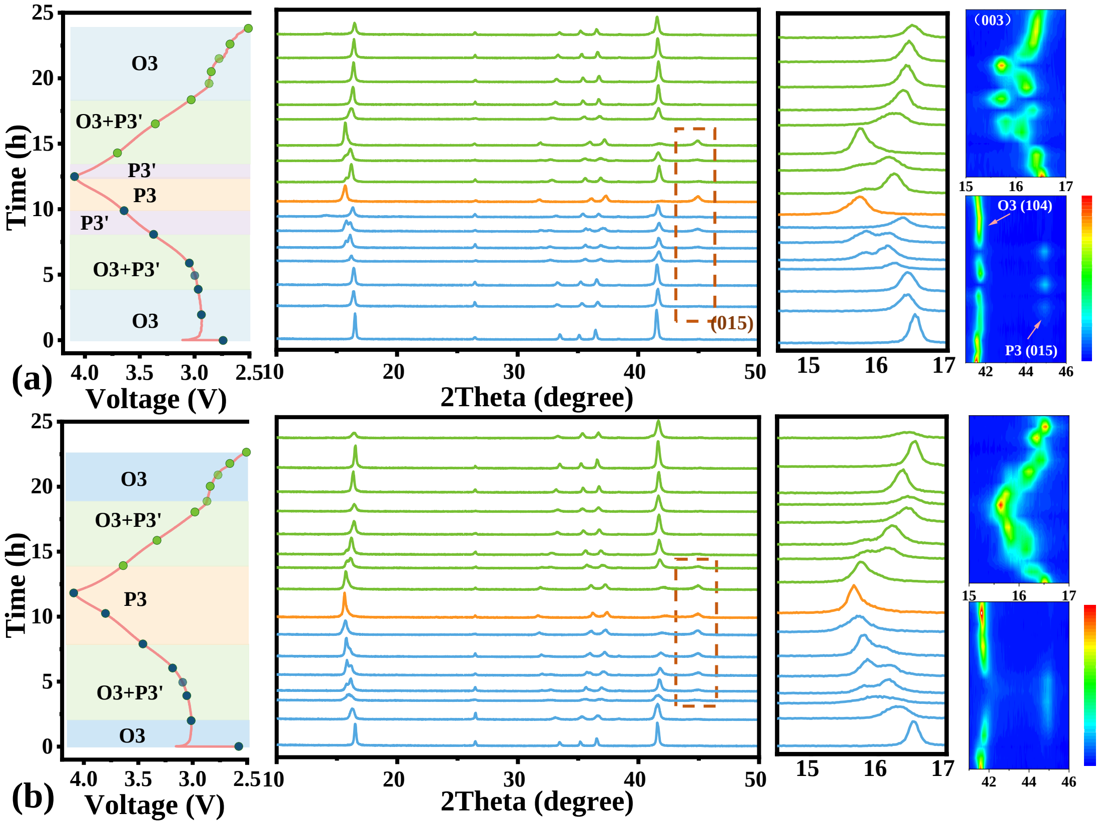

Figure S19 The structural evolution of NMF111 upon cycling. *ex-situ* XRD spectrum of (a) NMF111 and (b) 80%EA-0.1M-5min-NMF111 in the initial cycle. The selected points are Pristine, C2h, C4h, C5h, C6h, C8h, C10h, C4.1V, D2h, D4h, D6h, D7h, D8h, D9h, D10h, D2.5V, respectively.

Supplementary Note 10. The unstable phase change of NMF111 resulted from the gliding of the TM slab with the insertion/extraction of Na^{+}, while the Li^{+}-reinforced NMF111 behaved a smooth phase change between the O3 and P3 phases.

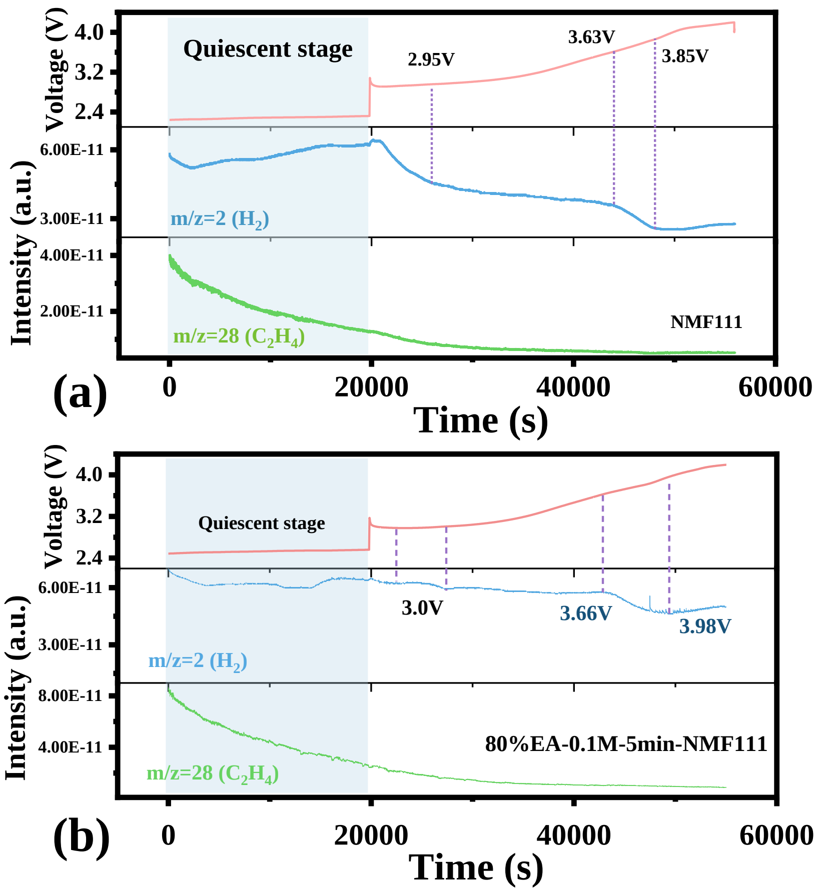

Figure S20 The gases emission inside the batteries upon charging. DEMS for the *operando* H_2 and C_2H_4 emission inside (a) NMF111//Na and (b) 80%EA-0.1M-5min-NMF111//Na half cells, respectively.

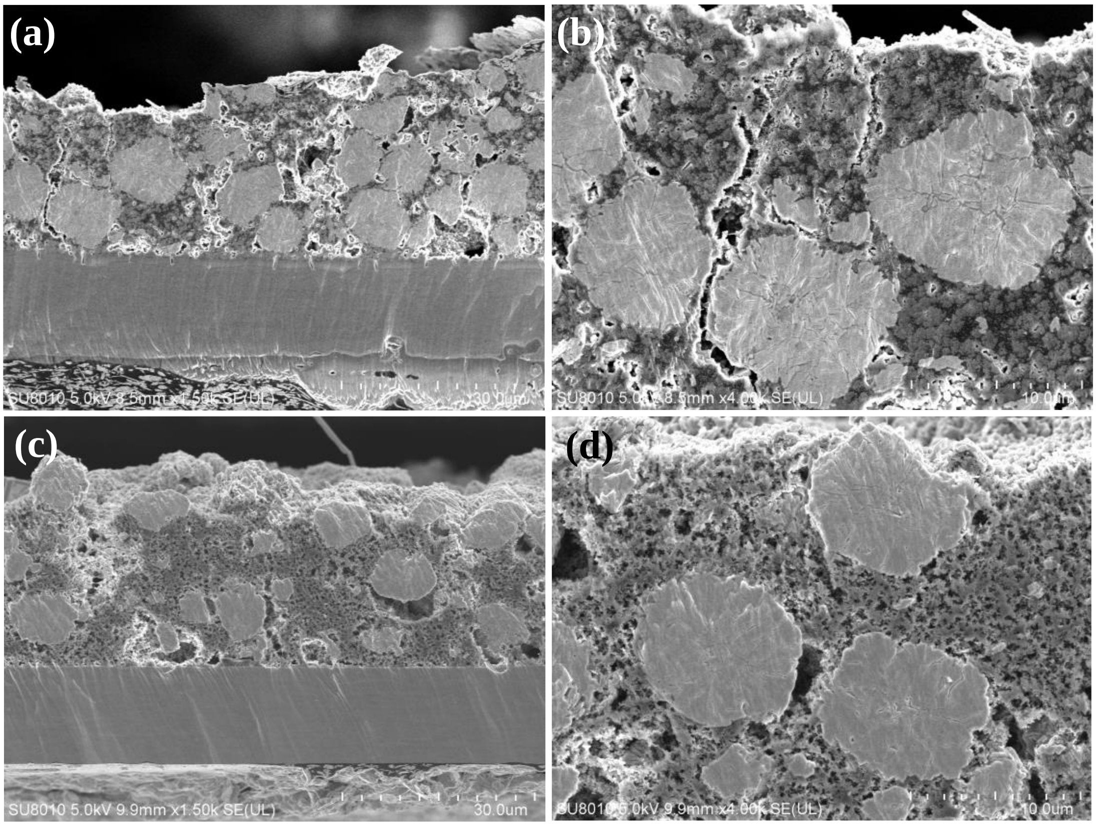

Figure S21 The cracks inside electrodes. The cross-section images of cycled (a, b) NMF111 and (c, d) 80%EA-0.1M-5min-NMF111 at 1C for 300 cycles.

Supplementary Note 11. It is obvious that stronger stress occurred in NMF111 after long cycling, which may result from the unstable phase change between the P3 and O3 phases.
